# Proceedings of the Chicago Medical Society

**Published:** 1868-08-01

**Authors:** 


### PROCEEDINGS OF THE CHICAGO MEDICAL SOCIETY.

SINCE the last report of the proceedings of this society, many instructive cases have been reported at its weekly meetings. During the summer the meetings will be held monthly. The following are are among the most interesting cases in which pathological specimens were presented:

*A case of placenta prævia, in which the fœtus was born with the membranes intact.*—Dr. Wanzer presented a fœtus of the eighth month, covered with the placenta and enraptured membrane, which had been delivered with the double complication of breech presentation, and placenta prævia. The patient had suffered from frequent hæmorrhages for two months previous to the delivery, and yet made a rapid recovery.

*Grey hepatization of the lungs not attended with cough.*—Dr. Durham exhibited the lungs of a female patient, who had for some months suffered from diarrhœa, abdominal pains and ascites. The diarrhœa was the first marked symptom preceded by considerable debility. There was absence of pain in the chest and cough.

The autopsy revealed not only tuberculous disease of the mesenteric glands, with peretonitis, but also most extensive gray hepatization of the lung, with purulent deposits.

*Singular fracture of the vertebra.*—Dr. Bogue presented the vertebra of a patient at the Cook County Hospital, who had fallen in front of a hand-car in rapid motion, so that the hand-car, in passing over, doubled him forward, producing a fracture of the last dorsal vertebra.

There was complete paralysis of sense and motion below the seat of fracture. The patient lost control over the evacuations of the rectum and bladder, and suffered from great prostration and enormous bed sores.

Death followed in thirty-seven days.

At the autopsy the body of the bone was found broken, the upper fragment having slipped forward and upward. The ribs were dislocated from their attachments. There was quite firm union of the bony tissue with absorption of the intervertebal substance.

The canal was still open, although very irregular at the point of fracture.

*Ciliary nerves.*—Dr. Holmes presented an eye which he had removed for a painful irido-choroiditis of thirteen months duration, the pupil being closed with lymph and the globe atrophied.

The specimen had been macerated two days in water, and then placed in a solution of bichromate of potash. It was specially interesting as presenting to view the minute branches and meshes of the ciliary nerve, as they lie embedded in the choroid. The choroid and sclerotic separated almost spontaneously on bisecting the globe, and yet were held together by the nerves at the points where they passed through the sclerotic. These minute nerves could be seen on slightly raising the choroid from the sclerotic. With a magnifying glass, the rich supply of nervous fibres in the ciliary bodies, could be distinctly seen.

*Removal of a large uterine tumor.*—Prof. Miller exhibited a large uterine tumor, of which he gave the following exceedingly interesting history:

Mrs. ——, a German woman, 23 years of age, was delivered of a child sixteen months ago. Eight months subsequent to this confinement she was seized with pains, and supposed she had a miscarriage, a midwife being in attendance. For a period of eight months she suffered from frequent attacks of hæmorrhage, pain in the abdomen, and considerable exhaustion. She consulted several physicians of well known skill and reputation, who diagnosticated the disease as inverted uterus.

Prof. Miller was first called to the case to reduce inversion. Upon making an examination, however, he discovered that the supposed inversion of the uterus was really a large tumo protruding from the os.

The growth was so long and its attachment so broad that it was almost impossible to introduce the sound into the cervix, without at the same time drawing down the tumor. If this was done, the sound could be readily passed nearly two inches and a half into the uterus.

Although the attachment of the tumor was very broad, there was little difficulty in applying the chain of the ecraseur, and removing the whole growth with scarcely any loss of blood.

The tumor was fibrous in character, measuring three inches in its long, and two in its short diameter.

The specimen presented an exception to the rule that such tumors are usually attached by narrow pedicles.

